# Sphingosine 1-phosphate receptor 2 mediated early stages of pancreatic and systemic inflammatory responses via NF-kappa B activation in acute pancreatitis

**DOI:** 10.1186/s12964-022-00971-8

**Published:** 2022-10-13

**Authors:** Jing Yang, Xujiao Tang, Baiqiang Li, Jinsong Shi

**Affiliations:** 1 School of Life Sciences and Health Engineering, Jiangnan University, Wuxi, Jiangsu China; 2 Department of Critical Care Medicine, Jinling Hospital, Nanjing University School of Medicine, Nanjing, Jiangsu China

**Keywords:** S1PR2, Acute pancreatitis, Inflammatory responses, Acinar cells, NF-kappa B

## Abstract

In acute pancreatitis, activation of inflammatory signaling, including the nuclear factor-kappa B (NF-κB) pathway, within acinar cells is known to be an early intracellular event occurring in parallel with pathologic trypsinogen activation. Sphingosine 1-phosphate receptor 2 (S1PR2) plays a critical role in endothelial inflammation, and our previous studies reported that S1PR2 deficiency significantly reduced the inflammatory response in liver injury under cholestasis conditions. However, the role of S1PR2 in inflammatory signaling activation within acinar cells and inflammatory responses during acute pancreatitis has not been elucidated. Here we report that S1PR2 was upregulated in the whole pancreas during acute pancreatitis. Blockade of S1PR2 by pharmacologic inhibition of S1PR2 by JTE-013 or AAV-mediated knockdown of S1PR2 improved the severity of pancreatic injury, as indicated by a significant reduction in inflammation and acinar cells death in acute pancreatitis mice. Moreover, S1PR2 is the predominant S1PRs expressed in pancreatic acinar cells and mediates NF-κB activation and the early inflammatory response within acinar cells under acute pancreatitis conditions via ROCK signaling pathways, not extracellular signal-regulated kinase pathways or p38 mitogen-activated protein kinase pathways. In addition, S1PR2 mediated macrophage NF-κB activation, migration and polarization toward the M1 phenotype. Therefore, these results demonstrated that the S1PR2-mediated early inflammatory response in acinar cells promotes the progression of acute pancreatitis, successfully linking local events to the systematic inflammatory response and leading to a novel therapeutic target for acute pancreatitis aimed at halting the progression of the inflammatory response.

**Video Abstract**

**Supplementary Information:**

The online version contains supplementary material available at 10.1186/s12964-022-00971-8.

## Introduction

Acute pancreatitis (AP) in both mild and severe forms, has high morbidity and mortality, and is a critical clinical problem worldwide [1, 2]. Despite recent significant advances, there remains a limited understanding of the pathogenesis of this disease, and the heterogeneity of disease presentations, and there is a lack of effective therapeutic options, with fluid resuscitation and supportive care as the only treatment for AP patients [3]. An increasing number of studies have focused on the sources and pathogenesis of the sterile inflammatory response in acute pancreatitis. Acinar cells are the major targets in acute pancreatitis [4]. As a critical role in the pathogenesis of pancreatitis, early activation of the inflammatory pathway in acinar cells leads to local inflammatory reactions and pancreatic injury. Once the local inflammatory reaction is uncontrolled, various proinflammatory mediators are released into the circulatory system, triggering a widespread inflammatory storm, progressively systemic inflammatory response syndrome (SIRS) and multiorgan dysfunction (MODS) [5–7]. Therefore, therapeutic targeting of early inflammatory events within acinar cells is a therapeutic option to prevent disease progression.

The nuclear factor-kappa B (NF-κB) pathway is the most widely studied inflammatory signaling pathway within acinar cells, and is correlated with a greater severity of acute pancreatitis [8]. NF-κB activation and trypsinogen activation are independent early events and play different roles in the development of acute pancreatitis [8–12]. NF-κB is present in the cytoplasm in an inactive form coupled to its inhibitor, IκB. Upon activation by infection or cellular stresses, IκB is phosphorylated, and degraded and releases NF-κB. Then, NF-κB translocates to the nucleus; and binds to specific elements located in the promoters of various genes, such as inflammatory factors, and adhesion molecules [13, 14].

Recent prominent research efforts have shown that sphingosine 1-phosphate (S1P)/S1P receptor (S1PR) signaling is a crucial inflammatory mediator and is involved in pathogenesis of various inflammatory-related diseases [15]. In endothelial cells, upregulation of S1PR2 activates the ROCK/NF-κB pathway and p38 MAPK, which are critical for the induction of vascular inflammation [16]. In human cholangiocarcinoma cells, activation of S1PR2 further promoted activation of NF-κB and subsequent COX-2 expression and prostaglandin E2 synthesis via the ERK1/2 and Akt signaling pathways [17]. Our previous study further indicated that in cholestasis-induced liver injury, S1PR2 promotes an inflammatory response and participates in disease progression [18]. Accumulating evidence has shown that S1PR2/3 activation recruits bone marrow-derived monocytes/macrophages to the damaged liver, promotes inflammatory M1 polarization and primes NLRP3 inflammasome and pro-inflammatory cytokine (IL-1β and IL-18) secretion, contributing to the progression of various liver injury progression [15, 19, 20]. Except for migration of bone marrow-derived monocytes/macrophages, which are different from neutrophils, cell migration and cytoskeletal remodeling were mediated by S1PR2. S1PR2 knockdown significantly lessened neutrophil infiltration and inhibited neutrophil extracellular rap (NET) formation, which ameliorated liver inflammation and fibrosis [21, 22]. Based on previous studies, S1PR2 is an interesting target for pancreatic systemic inflammatory responses during acute pancreatitis and is worth further study.

Here, we focused on exploring the influence of S1PR2 on the early inflammatory response within acinar cells under acute pancreatitis conditions. We found that S1PR2 is overexpressed in the pancreas, in acinar cells and macrophages, and inhibition of S1PR2 activation or knockdown of S1PR2 expression significantly inhibited acinar cell NF-κB activation, inflammatory cytokine release, macrophage recruitment and polarization toward the M1 phenotype. Finally, blockade of S1PR2 by JTE-013 also showed a protective effect against acute pancreatitis in vivo. In conclusion, these findings open new perspectives for S1PR2 as a target for the treatment of acute pancreatitis.

In the current study, we report that activation of S1PR2 plays an essential role in NF-κB activation in acinar cells and macrophages under acute pancreatitis conditions. Inhibition of S1PR2 activation using a specific chemical antagonist or knockdown of S1PR2 expression using a gene-specific shRNA significantly inhibited acinar cell NF-κB activation and macrophage migration and polarization toward the M1 phenotype. The chemical antagonist of S1PR2, JTE-013, and AAV-mediated knockdown of S1PR2 also showed protective effects against acute pancreatitis in vivo. Our study suggests that targeting S1PR2-mediated signaling pathways may have therapeutic potential for a subset of acute pancreatitis that currently lacks effective therapies.

## Materials and methods

### Materials

Caerulein was purchased from Nanjing peptide (China). LPS was purchased from Aladdin (China). Taurocholate acid (TCA) and sodium taurocholate were from Sigma-Aldrich (USA). JTE-013 was purchased from Cayman Chemicals (USA). The adeno-associated virus carrying the S1PR2-targeting shRNA or scramble control shRNA were obtained from KeyGEN Biotech Company (Obio Technology, China). The β-actin, S1PR2, p-ERK and T-ERK antibodies were purchased from Santa Cruz Biotechnology (USA). NF-κB(p65), RIP3 and cleaved-caspase 3 were purchased from Cell Signaling Technology (USA).

### Animals and treatment

All animal experiments were approved by the animal research committee of Jiangnan University and were performed in accordance with the principles of care and use of laboratory animals. ICR mice (male, 8 weeks) were purchased from Shanghai Slac Laboratory Animal Co. Ltd (China). For the caerulein pancreatitis model, caerulein (200 µg/kg) was intraperitoneally administered hourly ten times and LPS was intraperitoneally after the last caerulein injection, while the controls received identical PBS injections. For the taurocholate pancreatitis model, taurocholate acid was infused retrogradely into the pancreatic duct of mice and the controls received identical PBS injections. For the JTE-013 intervention groups, mice were intraperitoneally administered JTE-013 (10 mg/kg) 1 h before the first caerulein injection. After 12 h, serum and pancreatic tissue were harvested for further analysis (n = 5 per group). To construct the S1PR2 knockdown mouse model, mice were received intraperitoneal injection of adeno-associated virus carrying the S1PR2-targeting shRNA or scramble control shRNA. After 3 weeks, caerulein pancreatitis was induced in accordance with our previous study.

### Isolation of primary pancreatic acinar cells and primary peritoneal macrophages, cell culture and treatment

Pancreatic acinar cells and primary peritoneal macrophages were acquired from mice aged 8 weeks as previously described [23, 24]. Mouse pancreatic acinar carcinoma 266-6 cells were obtained from Cobioer Biosciences Co, Ltd. and mouse macrophages RAW264.7 were obtained from American Type Culture Collection. All cells were cultured in DMEM with 10% FBS, 100 U/ml penicillin and 100 mg/ml streptomycin.

### Western blot analysis

The pancreas and cell samples were homogenized in RIPA lysis buffer, and total proteins were isolated and analyzed by western blot using specific primary and secondary antibodies as previously described [25]. An ECL chemiluminescent kit was used to visualize the immunoreactive bands, and ImageJ software was used to analyze the density of immunoreactive images.

### Immunofluorescence staining

Fresh pancreatic tissues were fixed, sliced, dewaxed, rehydrated, subjected to antigen retrieval, blocked with 5% BSA and stained for S1PR2.

For cell samples, cells were plated on coverslips, cultured overnight and treat with TCA (100 µM), in the presence or absence of JTE-013 (10 µM) for 4 h. Cells were fixed with 4% paraformaldehyde, permeabilized with 0.2% Triton X-100, blocked with 5% BSA for 1 h, incubated with NF-κB (p65) antibody (1:200 dilution) overnight at 4 °C and incubated with Alexa Fluor 488 secondary antibody for 1 h, and finally filmed with the anti-fluorescence quencher DAPI. Images were recorded using a Carl Zeiss LSM880 microscope.

### Real-time PCR

Extraction of total RNA from cells and real-time RT-PCR were performed as described previously [25]. The mRNA levels of *S1PR*s, *IL-6, TNF-α, CD86, arginase, CD206*, and *CD163* were determined. All data were normalized to β-actin as an internal control.

### Macrophage migration assay

RAW264.7 cells were seeded in the upper chamber and treated with TCA (100 µM), in the presence or absence of JTE-013 (10 µM) for 24 h, while the chemoattractant (CCL2, 100 ng/mL) was added to the lower compartments. The migrated cells on the lower surface of the chamber were stained with crystal violet solution and detected by light microscopy as described previously [26].

### RNA interference

Cells were cultured overnight to 50% confluence and transiently transfected with control shRNA or S1PR2 shRNA. After 48 h, the cells were used for further experiments.

### Measurement of serum lipase and α-amylase activity

Blood from mice was collected and centrifuged (4000 rpm, 10 min, 4 °C) to obtain the supernatant for further detection. The serum activities of α-amylase and lipase were detected using assay kits (Jiancheng Biotech, China). All kits were used according to the manufacturer’s instructions.

### HE staining

Fresh pancreases were fixed with 4% paraformaldehyde, and embedded with paraffin for H&E staining and examined by light microscopy.

### Immunohistochemistry (IHC) staining

Fresh pancreases were fixed, sliced, dewaxed, rehydrated, subjected to antigen retrieval, blocked with 5% BSA and staining for Ly6G and CD68. Finally, images were captured with a light microscope.

### Enzyme-linked immunosorbent assays (ELISA)

Serum TNF-α and IL-6 levels were measured using enzyme-linked immunosorbent assay (ELISA) kits (Proteintech, China) according to the manufacturer’s protocols.

### Statistical analysis

The results are expressed as the mean ± SEM. Statistical significance among multiple groups was determined using one-way variance followed by Bonferroni's multiple comparison test. Statistical analyses were performed using GraphPad Prism 8 software, and *p* < 0.05 was considered statistically significant.

## Results

### Overexpression of S1PR2 in the pancreas of mice with acute pancreatitis

**Fig. 1:**
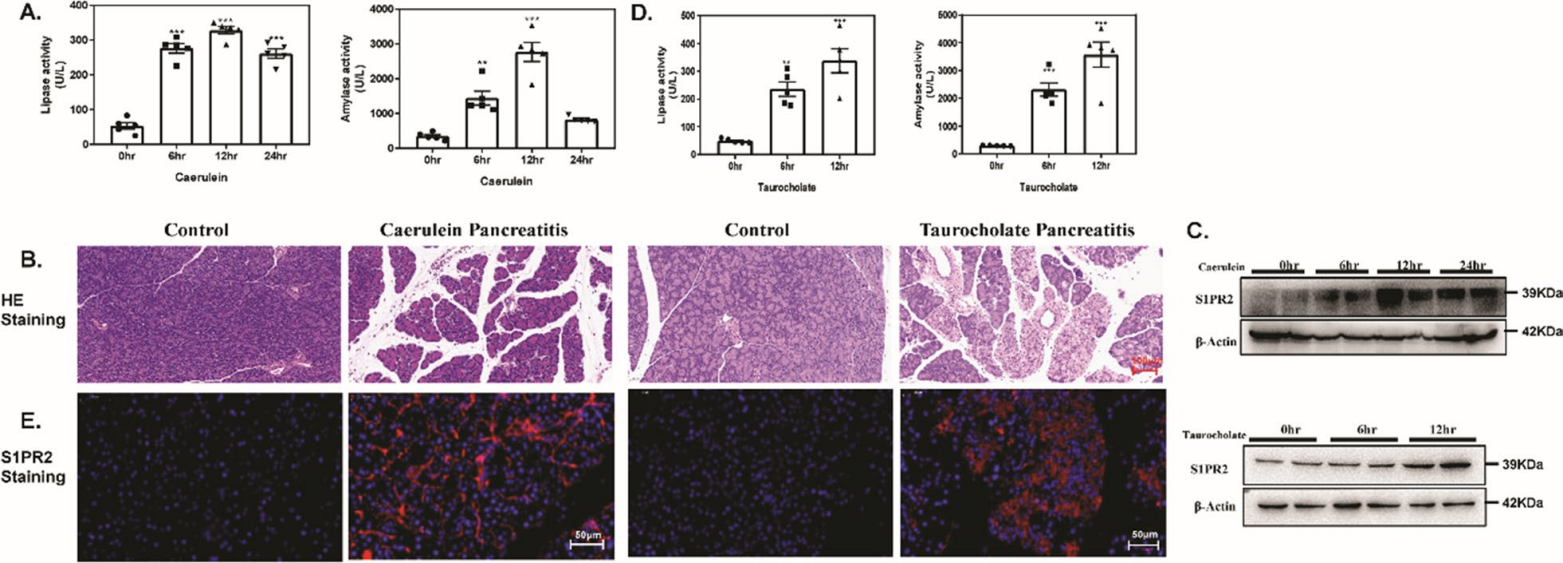
Overexpression of S1PR2 in the pancreas of acute pancreatitis mice. Serum lipase and serum amylase (**A**) levels in caerulein pancreatitis; pancreatic H&E staining in caerulein pancreatitis and taurocholate pancreatitis mice (**B**); western blotting of S1PR2 protein levels in the pancreas of caerulein pancreatitis and taurocholate pancreatitis mice (**C**); serum lipase and serum amylase (**A**) levels in taurocholate pancreatitis (**D**); immunofluorescence staining of pancreatic S1PR2 expression (**E**). Data shown are means ± SEM. ***p < 0.001 compared with the control group

Caerulein-induced acute pancreatitis (caerulein pancreatitis) and retrograde pancreatic duct injection of taurocholate induced severe necrotizing pancreatitis (taurocholate pancreatitis) are two widely used in vivo experimental acute pancreatitis models [27]. We examined the expression of S1PR2 in these two experimental acute pancreatitis models. In the caerulein pancreatitis model, after 6, 12 and 24 h of repeated caerulein treatment, serum lipase and amylase levels were upregulated in a gradual manner, which revealed that exposure to caerulein resulted in pancreatic damage (Fig. 1A). In addition, the acinar cell necrosis, interlobular space, and inflammatory cell infiltration was significantly increased after caerulein treatment (Fig. 1B). Caerulein-induced injury significantly stimulated S1PR2 expression at the protein levels after 12 h (Fig. 1C). Similar to the findings in taurocholate pancreatitis, the lipase and amylase levels in the serum increased to some degree (Fig. 1D), and extensive acinar necrosis occurred, with mast inflammatory cells infiltrated in the taurocholate group (Fig. 1B), indicating the pancreas was greatly impacted. Western blot analysis showed that S1PR2 was markedly increased at 12 h in the pancreas of taurocholate pancreatitis model mice compared to the control (Fig. 1C). In addition, the immunofluorescence results indicated that positive S1PR2 staining was obviously upregulated in acute pancreatitis mice by either caerulein or taurocholate, and most of the anti-S1PR2 staining is localized at the acinar cells (Fig. 1E). The above results indicated that S1PR2 was overexpressed in the pancreas during acute pancreatitis.

### Pharmacological inhibition of S1PR2 by JTE-013 alleviates the severity of pancreatic injury in mice with acute pancreatitis

**Fig. 2:**
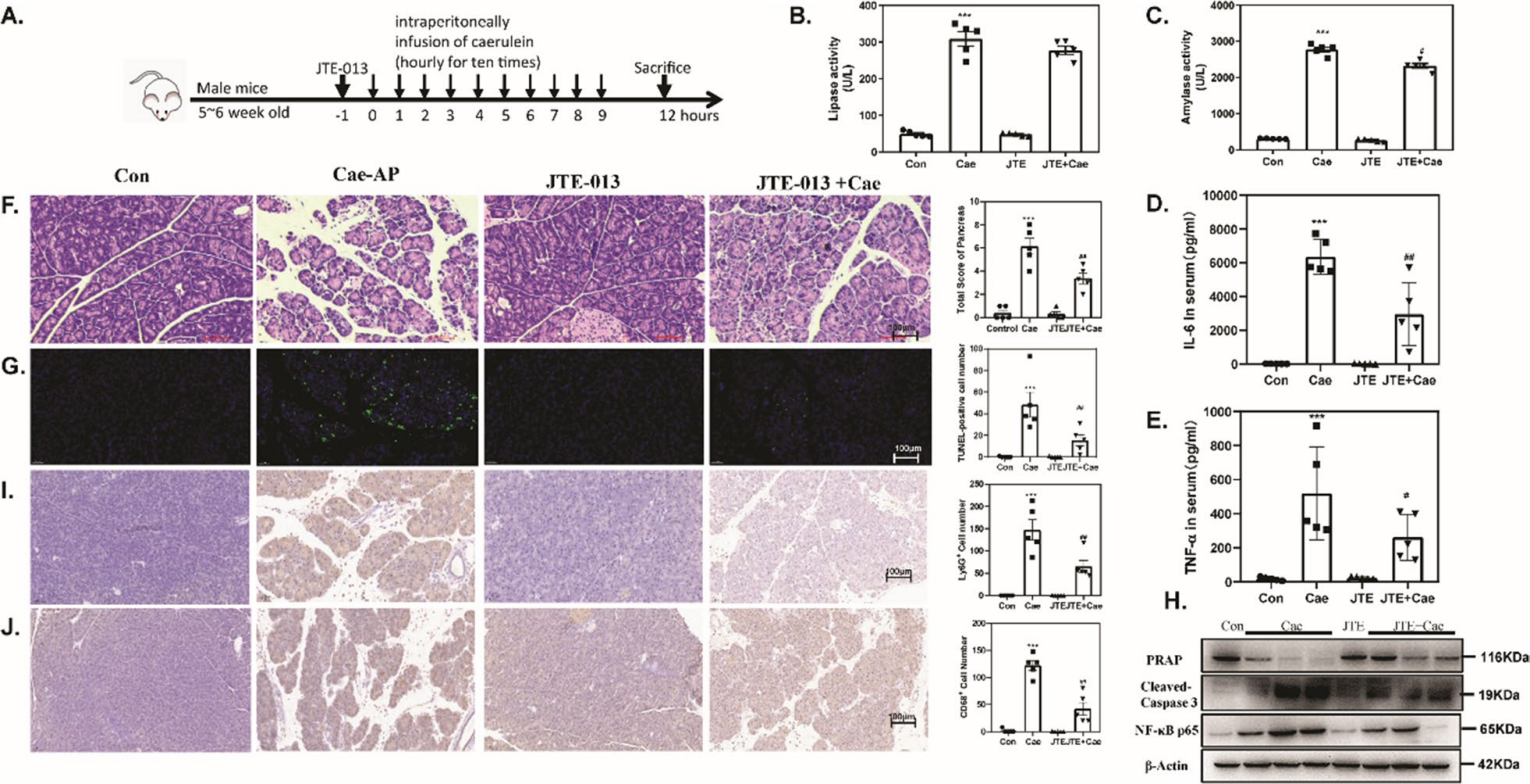
Pharmacological inhibition of S1PR2 by JTE-013 alleviates the severity of pancreatic injury in acute pancreatitis mice. Schematic representation of the experimental schedule (**A**); serum lipase (**B**) and serum amylase (**C**) levels; ELISA of serum IL6 (**D**) and TNF-α (**E**); H&E-staining of pancreas sections (**F**); TUNEL staining(**G**); Western blot of pancreatic PARP, cleaved-caspase3 and NF-κB p65 (**H**) levels in the pancreas; Ly6G staining (**I**); CD68 staining (**J**). Data shown are means ± SEM. ***p < 0.001 compared with the control group. ##p < 0.05 compared with the model group

The significant increase in S1PR2 expression in the acute pancreatitis model prompted us to elucidate the function of S1PR2 in acute pancreatitis. First, we examined whether blockade of S1PR2 expression could affect the initiation and progression of acute pancreatitis. The specific antagonist of S1PR2, JTE-013, was injected to mice 1 h before acute pancreatitis induction (Fig. 2A). Twelve hours after the caerulein challenge, the pancreas injury index, including serum lipase, α-amylase (Fig. 2B, C) and inflammatory cytokines, including IL-6 and TNF-α (Fig. 2D, E), were significantly upregulated in mice induced with caerulein pancreatitis but decreased significantly after JTE-013 intervention. Consistent with the changes in pancreatic function, HE staining of pancreas sections revealed that exposure to caerulein resulted in extensive pancreatic edema, inflammatory infiltration and local necrosis, and JTE-013 treatment significantly attenuated the histological features of pancreatic injury (Fig. 2F).

In addition, the TUNEL-positive acinar cells significantly increased in response to caerulein supramaximal stimulation, as expected, while it was markedly reduced in the JTE-013 intervention group (Fig. 2G). Furthermore, we verified the influence of JTE-013 on cell apoptosis markers, cleaved-caspase 3 and poly ADP-ribose polymerase (PRAP) in caerulein pancreatitis. After caerulein stimulation, cleaved-caspase 3 and PRAP protein expression levels in pancreatic tissue were dramatically increased or decreased (Fig. 2H). Meanwhile, JTE-013 administration successfully abrogated the expression of these death markers compared to that in caerulein pancreatitis mice (Fig. 2H).

Immunohistochemistry results revealed that Ly6G + neutrophils and CD68 + macrophages were abundantly recruited in the caerulein pancreatitis group as compared to the normal control group. After JTE-013 administration, these was a significant reduction in the number of Ly6G + neutrophils and CD68 + macrophages in the pancreas (Fig. 2I, J. Western blotting also suggested that JTE-013 administration reversed the increase in NF-κB p65 protein expression in acute pancreatitis (Fig. 2H). These results indicated that blockade of S1PR2 by JTE-103 efficiently inhibited inflammatory cell recruitment and infiltration and abated the pancreatic inflammatory response, confirming that S1PR2 inhibition was protective against acute pancreatitis.

### S1PR2 knockdown obviously alleviates pancreatic damage in acute pancreatitis mice

**Fig. 3:**
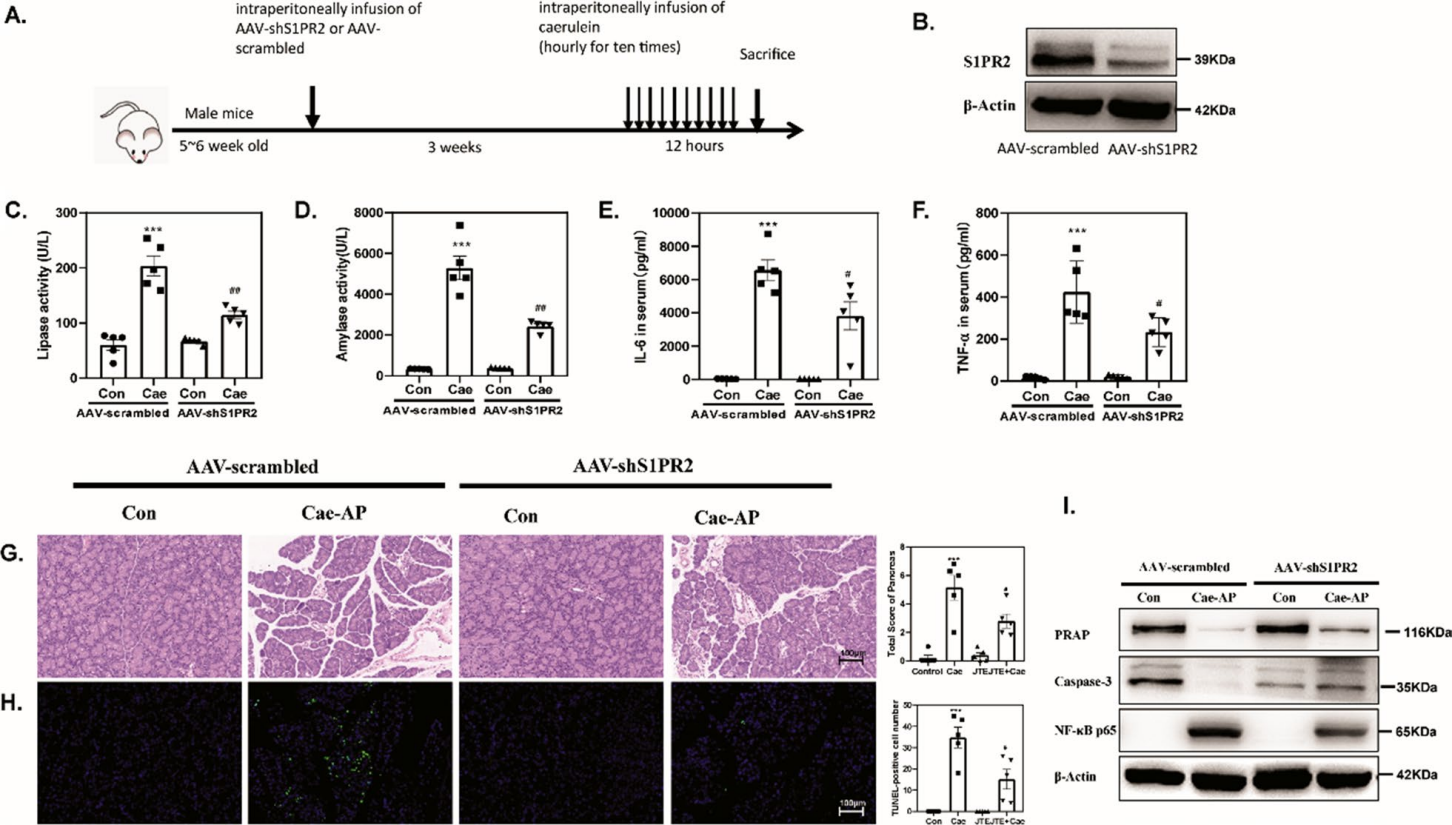
S1PR2 knockdown alleviates pancreatic damage in acute pancreatitis mice. Schematic representation of the experimental schedule (**A**); western blot of pancreatic S1PR2 (**B**); serum lipase (**C**) and serum amylase (**D**) level; ELISA of serum IL6 (**E**) and TNF-α (**F**); H&E-staining pancreas sections (**G**); TUNEL staining (**H**); Western blot of pancreatic PARP, caspase3 and NF-κB p65 (**I**) levels in the pancreas. Data shown are means ± SEM. ***p < 0.001 compared with the control group. #p < 0.01, ##p < 0.05 compared with the model group

To determine whether S1PR2 mediated sustained pathological inflammation in vivo, we subjected mice to intraperitoneal injection of S1PR2-shRNA (Fig. 3A) S1PR2-shRNA markedly and specifically downregulated S1PR2 expression in the pancreas of acute pancreatitis mice (Fig. 3B). S1PR2 blockade significantly decreased the levels of lipase and α-amylase (Fig. 3C, D) and the concentrations of inflammatory cytokines including IL-6 and TNF-α (Fig. 3E, F) in the serum of the injured mice. Simultaneously, histologic analysis showed that the interlobular space, acinar necrosis and inflammatory cell infiltration were predominantly attenuated under S1PR2-shRNA administration (Fig. 3G, H). Moreover, acinar cell death hallmarks (PARP, and Caspase3) and NF-κB activation were obviously reduced in the injured pancreas (Fig. 3I). Taken together, these results prove that S1PR2 knockdown may be an excellent therapeutic strategy for acute pancreatitis via alleviating pancreatic injury.

### S1PR2 is the predominant s1prs expressed in pancreatic acinar cells

**Fig. 4:**
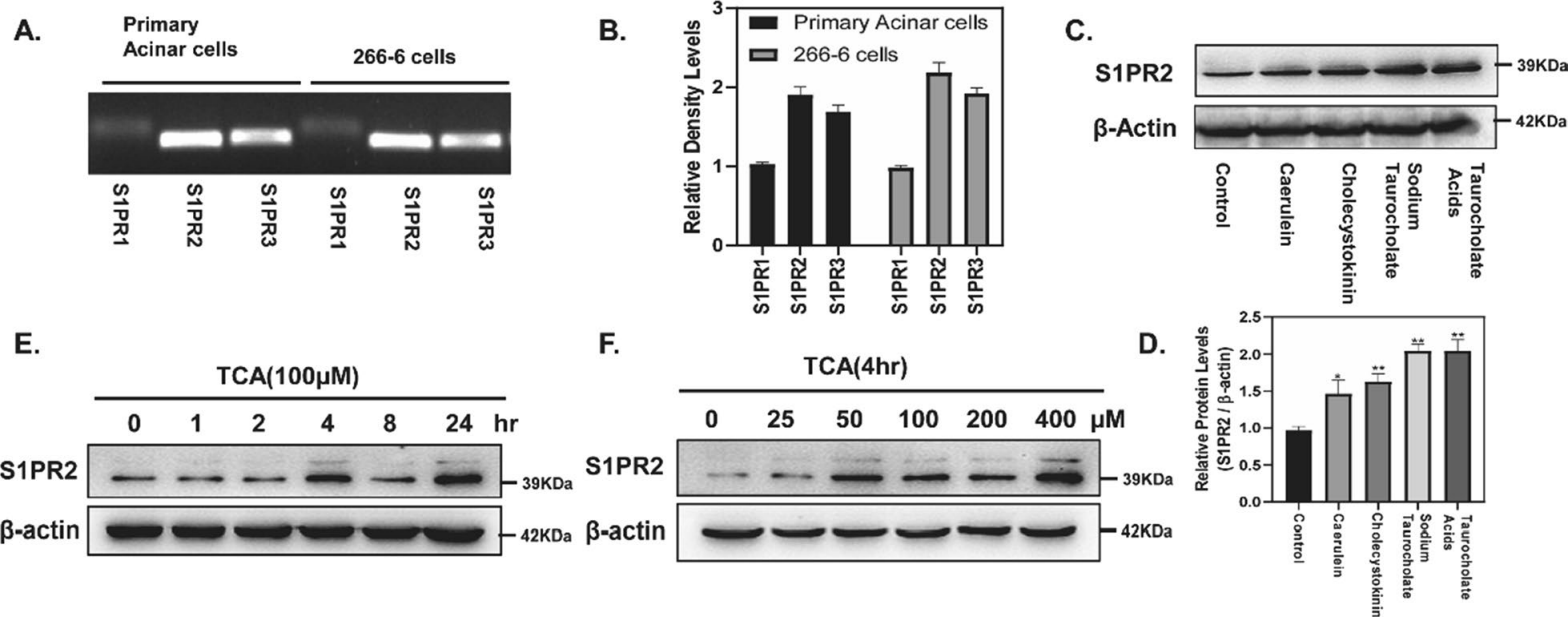
S1PR2 is the predominant S1PR expressed in pancreatic acinar cells. S1PR mRNA expression in primary acinar cells and 266-6 cells (**A**, **B**); S1PR2 expression in pancreatic acinar cells during acute pancreatitis in vitro (**C**, **D**); S1PR2 expression in 266-6 cells stimulated for different times (E) and different doses (F) of TCA

Primary pancreatic acinar cells and acinar carcinoma cells 266-6 (mouse) are often used in vitro experimental AP models. PCR analysis demonstrated that S1PR2 was the predominant S1PR expressed in pancreatic acinar cells and 266-6 cells (Fig. 4A, B). Figure 4C, D illustrated that S1RP2 was up-regulated in primary pancreatic acinar cells after stimulated with caerulein, cholecystokinin, sodium taurocholate or taurocholate acid (TCA). In 266-6 cells, TCA can induced high S1PR2 expression in a time- and dose- dependent manner (Fig. 4E, F).

### NF-κB activation and inflammatory factor expression in pancreatic acinar cells induced by TCA

**Fig. 5:**
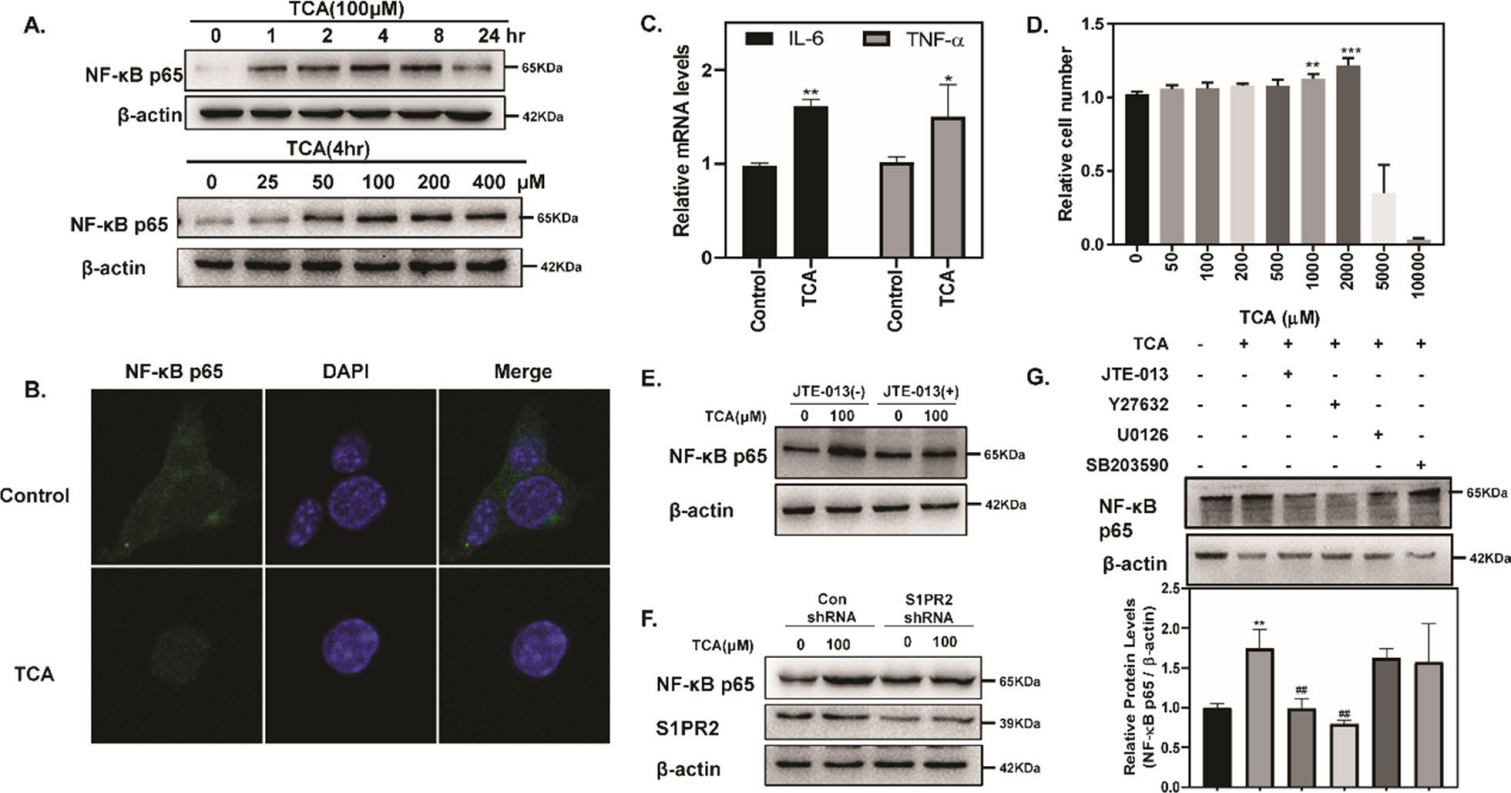
S1PR2 mediated NF-κB activation and inflammatory factor expression in pancreatic acinar cells. NF-κB expression in 266-6 cells (**A**); immunofluorescence staining of NF-κB in 266-6 cells (**B**); mRNA levels of the inflammatory factor IL-6 and TNF-α in 266-6 cells (**C**); cell proliferation in 266-6 cells (**D**); NF-κB levels in 266-6 cells, preincubated with or without JTE-013, and stimulated with TCA (100 µM) for 4 h (**E**); S1PR2 and NF-κB levels in adenovirus control and Ad-S1PR2-transduced 266-6 cells stimulated with TCA (100 µM) for 4 h (**F**); NF-κB levels in 266-6 cells, preincubated with or without JTE-013, Y27632, U0126, SB203590, and stimulated with TCA (100 µM) for 4 h (**G**). Data shown are means ± SEM. *p < 0.01, **p < 0.05, ***p < 0.001 compared with the control group. ##p < 0.05 compared with the TCA-treated alone

Cytokines and inflammatory signaling play a critical role in the progression of acute pancreatitis, and are not only released by infiltrating inflammatory cells but also by acinar cells [5]. Due to the short in vitro viability of primary pancreatic acinar cells, we used 266-6 cells to assess the activation of NF-κB. The results showed that TCA upregulated the protein level of NF-κB p65 in both a time- and dose- dependent manner (Fig. 5A). Moreover, TCA significantly increased NF-κB p65 nuclear translocation (Fig. 5B). Consistent with NF-κB activation, there was a marked elevation in the expression of the NF-κB target genes IL-6 and TNF-α by TCA stimulation (Fig. 5C). In addition, we used the CCK-8 method to evaluate the cytotoxic effect of TCA in 266-6 cells, and the TCA dose we used (100 nM) did not have a cytotoxic effect (Fig. 5D), which hinted that TCA induced the activation of NF-κB independent of its cytotoxic effect.

### S1PR2 mediates NF-κB activation induced by TCA in pancreatic acinar cells

To examine whether S1PR2 is responsible for inflammatory response within acinar cells during the early phase of pancreatitis, we treated cells with JTE-013 (an antagonist of S1PR2) or S1PR2-shRNA to block the effects. As shown in Fig. 5E, F both inhibition of S1PR2 activation by JTE-013 and knockdown of S1PR2 expression by S1PR2-shRNA reversed TCA-induced NF-κB activation, further suggesting that TCA activates NF-κB via activation of S1PR2 in pancreatic acinar cells.

S1PR2 is a G protein-coupled receptor and has various biological functions by activating multiple downstream signaling pathways, such as the Rho kinase (ROCK), ERK, and p38 signaling pathways [28]. Next, we used pharmacological inhibitors of signal transduction, including Y27632 (ROCK inhibitor), U0126 (ERK inhibitor), and SB203580 (p38 inhibitor), to dissect the signal pathways responsible for NF-κB activation in acinar cells regulated by TCA. The TCA-induced increase in NF-κB protein level was only lessened by Y27632 in mouse 266-6 cells, whereas U0126 and SB203580 had no effect (Fig. 5G), indicating that ROCK signaling, not ERK or p38 signaling, might be necessary for TCA-induced NF-κB activation in pancreatic acinar cells.

### S1PR2 is the predominant S1PR expressed in macrophages and mediates NF-κB activation in macrophages

**Fig. 6:**
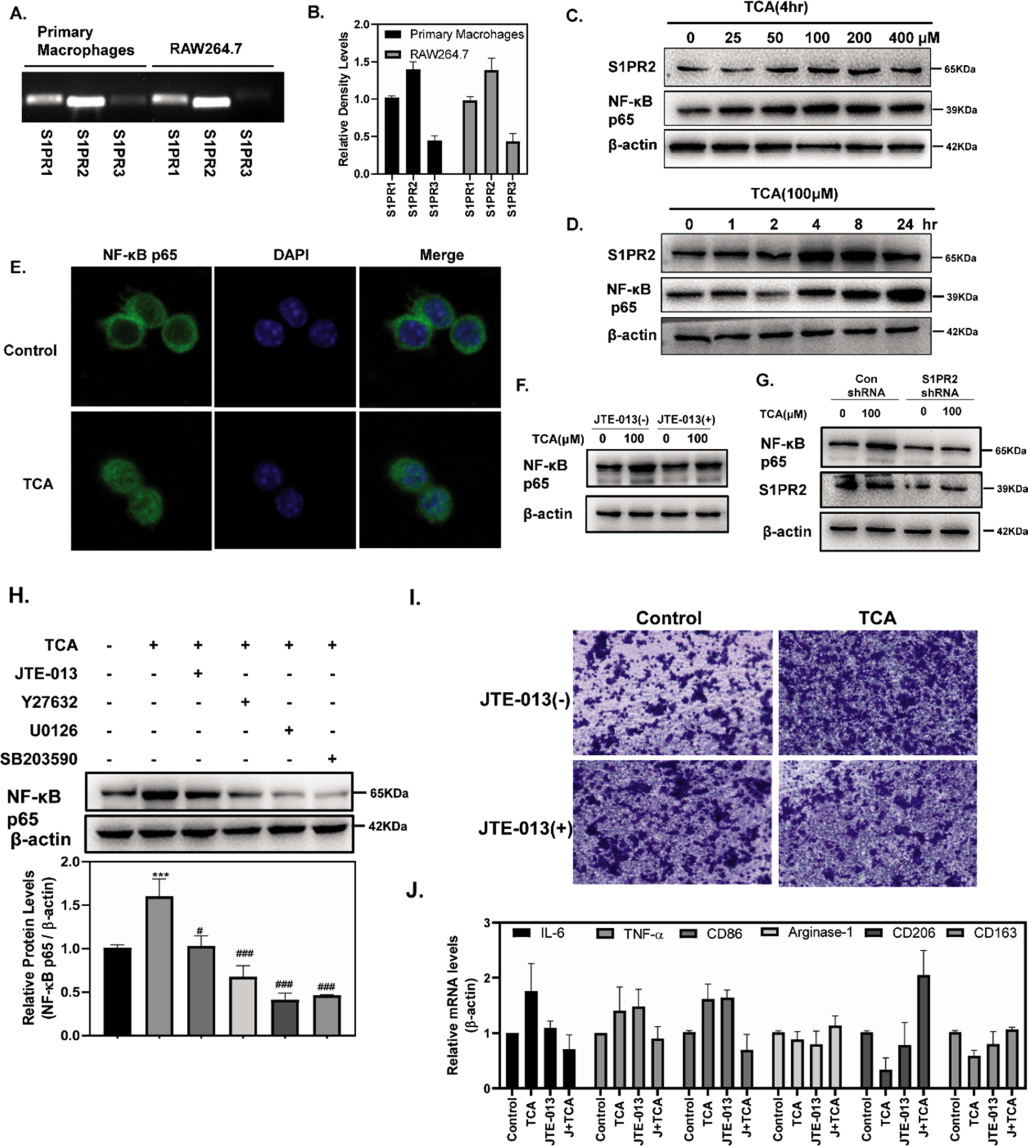
S1PR2 is the predominant S1PR expressed in macrophages and mediates NF-κB activation in macrophages. mRNA expression of S1PRs in primary peritoneal macrophages and RAW264.7 cells (**A**, **B**); S1PR2 and NF-κB p65 levels in RAW264.7 cells stimulated by different times and doses of TCA (**C**, **D**); Immunofluorescence staining of NF-κB in RAW264.7 cells (**E**); NF-κB levels in 266-6 cells, preincubated with or without JTE-013 and stimulated with TCA (100 µM) for 4 h (**F**); S1PR2 and NF-κB levels in adenovirus control and Ad-S1PR2–transduced RAW264.7 cells stimulated with TCA (100 µM) for 4 h (**G**); NF-κB levels in RAW264.7 cells, preincubated with or without JTE-013, Y27632, U0126, SB203590, and stimulated with TCA (100 µM) for 4 h (**H**); RAW264.7 cells were cultured in transwell inserts, and stimulated with JTE-013 for 30 min and then treated with TCA (100 µM) for 24 h (**I**); The mRNA levels of inflammatory factors in RAW264.7 cells stimulated with TCA (100 µM) for 4 h with or without JTE-013 (**J**). Data shown are means ± SEM. ***p < 0.001 compared with the control group. #p < 0.01, ###p < 0.001 compared with the TCA-treatment alone

Macrophages are the prominent infiltrating cells in early pancreatitis and is critical in the widespread systemic inflammatory response [29]. Studies have shown that S1PR2 is highly expressed in macrophages and macrophage S1PR2 retards liver inflammation and fibrogenesis [19]. We analyzed the expression of S1PR2 in isolated primary peritoneal macrophages and RAW264.7 cells in acute pancreatitis models. The PCR results demonstrated that S1PR2 was the predominant S1PR expressed in primary peritoneal macrophages and RAW264.7 cells (Fig. 6A, B). Figure 6C, D revealed that TCA can induced high S1PR2 expression in RAW264.7 cells in a dose- and time-dependent manner. Figure 6C, D revealed that TCA upregulated protein level of NF-κB p65 in both a dose- dependent and time-dependent manner. TCA also significantly increased the nuclear translocation of NF-κB p65 (Fig. 6E). To identify whether S1PR2 induced NF-κB activation in macrophages, JTE-013 or S1PR2 shRNA was used in subsequent experiments. Figure 6F, G indicated that TCA significantly increased the protein levels of NF-κB p65, and this was reversed by both JTE-013 and S1PR2 shRNA. Next, the increase in NF-κB protein levels was lessened by Y27632, U0126 and SB203580 in mouse 266-6 cells (Fig. 6H), indicating that ROCK, ERK and p38 signaling might be necessary for NF-κB activation in macrophages.

### Activation of S1PR2 promotes macrophage migration and macrophage polarization toward the M1 phenotype

To verify the effects of S1PR2 activation on macrophage migration, RAW264.7 cells were seeded in the upper chamber of a transwell insert and treated with TCA (100 µM) or vehicle control in the presence or absence of JTE-013. The number of migrating cells in the TCA group was markedly increased compared to that in the control group, which were obviously blocked by JTE-013 (Fig. 6I).

Additionally, we showed that TCA treatment upregulated the expression of M1 macrophage marker genes, including IL-6, TNF-α, and CD86, while it downregulated the expression of M2 macrophage marker genes, including arginase, CD206, and CD163 (Fig. 6J). Moreover, JTE-013 treatment significantly downregulated the expression of M1 marker genes, and particularly promoted the expression of M2 marker genes, which further suggested that activation of S1PR2 promotes macrophage polarization toward the M1 phenotype.

## Discussion

We previously revealed that S1PR2 promotes an inflammatory response and participates in the progression of cholestasis-induced liver injury. In the present study, we identified a critical role of S1PR2 in acute pancreatitis. Pharmacologic inhibition of S1PR2 signaling by JTE-013 or knockdown of S1PR2 expression using a gene-specific shRNA alleviated pancreatic injury and resulted in lower inflammatory responses in caerulein pancreatitis mouse models. Importantly, the protective mechanisms depend on the inhibition of S1PR2 on acinar cells and macrophages, which altered the early activation of inflammatory pathways, including NF-κB activation in the acinar cells, decreased peritoneal macrophage recruitment and regulated the M1 phenotype polarization of macrophages.

Despite significant advances in the last 25 years, the mechanisms responsible for the initiation of acute pancreatitis remain elusive. Acute pancreatitis is attributed to premature intracellular trypsinogen activation initiating pancreatic acinar cell injury, and releasing various DAMPs or cytokines, which lead to inflammatory cell recruitment into the damaged pancreas [30, 31]. However, in vitro expression of active trypsin cannot activate NF-κB, which suggests that trypsinogen activation and NF-κB activation are independent events during the early phase of acute pancreatitis. Under conditions of stress, including alcohol abuse, cigarette smoking, or hyperlipidemia, acinar cells show such unique inflammatory properties [32]. Thus far, studies using transgenic animals in acute pancreatitis, such as T7-/- and Ctsb-/- mice, have successfully identified that activation of NF-κB pathway within acinar cells is a key early event in pancreatitis and may have a hand in the progression of pancreatic and systemic inflammatory responses [30]. In the current study, TCA induces NF-κB upregulation and translocation to the nucleus in pancreatic acinar cells in both a dose- and time- dependent manner, and these effects were blocked by both JTE-013 (an antagonist of S1PR2) or S1PR2-shRNA, which further showed that S1PR2 activation was responsible for inflammation within acinar cells during the early phase of pancreatitis. Consistent with our reports, upregulation of S1PR2 activates the NF-κB signaling pathway and is critical for vascular inflammation induction.

Notably, monocytes/macrophages, but not NK, NKT, or TCRγδ + T cells, participate in the early stage of the pathogenesis of caerulein pancreatitis and emerge at the highest ratio among the immune cells in the damaged pancreas of caerulein-injected mice [33, 34]. In this study, one striking finding was that TCA promoted macrophage migration and regulated macrophage polarization toward the M1 phenotype via S1PR2 in vitro. As expected, administration of the S1PR2 antagonist JTE-013 resulted in a lower number of infiltrating macrophages and lower levels of inflammatory factors during acute pancreatitis in vivo. Emerging evidence indicates that S1PR2 participates in neutrophil recruitment and NETosis, another form of activated neutrophil death, during BDL-induced liver injury [22]. We also demonstrated that blockade of S1PR2 alleviated Ly6G + neutrophil infiltration in acute pancreatitis mice.

**Fig. 7:**
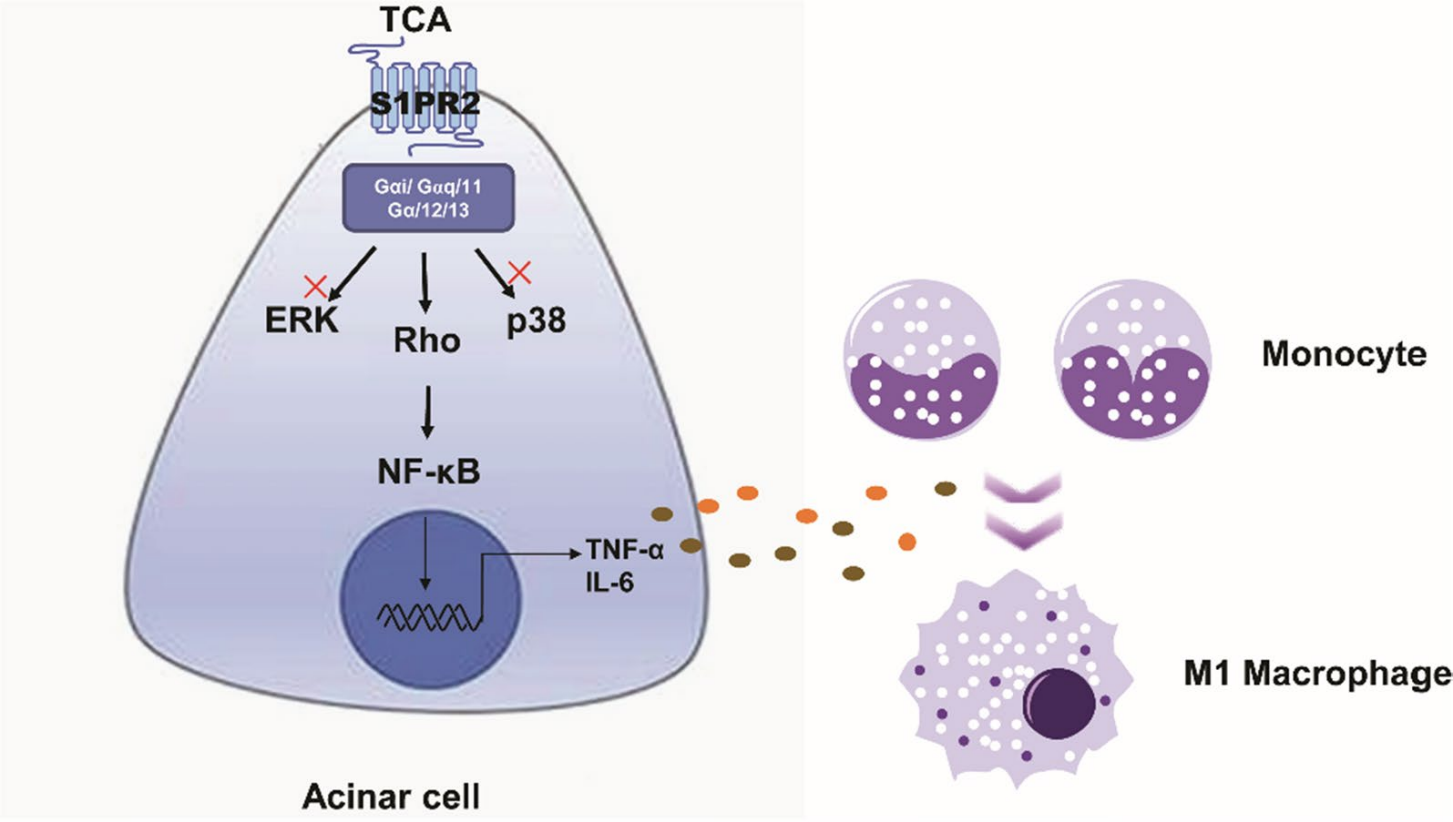
Schematic diagram: S1PR2 mediates early activation of inflammatory pathways, particularly though NF-kappa B activation in acinar cells, recruits macrophages, promotes macrophages polarization toward the M1 phenotype, and participates in pancreatic local and systemic inflammatory responses in acute pancreatitis

S1PR2 is a G protein-coupled receptor and couples with several different G-alpha subunits, such as Gα_(i/o)_, Gα_q_, and Gα_(12/13)_ [28, 35]. Activation of these subunits exert different influences on various pathways, due to the different stimuli and different cell type [36]. Here, we further evaluated which downstream second messenger molecules of S1PR2, such as Rho kinase (ROCK), ERK and p38 MAPK, are responsible for NF-κB signaling pathway activation in pancreatic acinar cell. The results showed that TCA-induced NF-κB activation and inflammatory factor secretion were impaired by ROCK inhibitors in pancreatic acinar cells, while ERK and p38 inhibitors had no such effect. Nevertheless, in macrophages, ROCK, ERK, and p38 inhibitors impaired TCA-induced NF-κB activation. Consistent with our results, S1PR2 predominantly couples to the Rho/ROCK pathway and leads to NF-κB activation in macrophages. Subsequently, blockade of S1PR2 activates the Rho/Rho kinase/NF-κB signaling pathway and reduces cytokine secretion and oxidized LDL uptake [16, 37]. Finally, homology modeling of S1PR2 docking to TCA predicts that TCA, a low affinity agonist, hydrogen bonds only to Leu 173 of S1PR2 [38]. Overall, this study highlighted that S1PR2 is the critical role in acute pancreatitis. In pancreatic acinar cells, activation of S1PR2 by TCA promotes NF-κB transactivation through the ROCK signal pathway, leading to the secretion of inflammatory factors, further recruiting immune cell, especially macrophages, and promoting M1 phenotype polarization during acute pancreatitis (Fig. 7). Furthermore, our results support that S1PR2 blockade can alleviate the early inflammatory response and provide new compelling therapeutic information for acute pancreatitis.

## Data Availability

The data that support the findings of this study are available in the “Methods” section of this article.
